# Supplementary material for: Concordance between self-report and six commonly used clinical estimates or serological measures: Insights from a Canadian healthy aging study
Source: PLoS One. 2026 Apr 8;21(4):e0346489. doi: 10.1371/journal.pone.0346489 (PMC13061170; doi:10.1371/journal.pone.0346489)
Supplement: S1 Appendix — (DOCX) [file pone.0346489.s001.docx]

**S1 Appendix.** Demographic characteristics of women living with HIV and without HIV in the BCC3 study

|  | **Women living with HIV**  ***n* = 220** | **Women without HIV**  ***n* = 309** | ***p*-value** |
| --- | --- | --- | --- |
| **% of total sample** | 41.6 | 58.4 |  |
| **Age,** years, *median (IQR)* | 49 (41-58) | 46 (34-57) | 0.008^a^ |
| **Ethnicity**, *n* (%)    white    Indigenous    African/Caribbean/Black    Other racialized groups    Missing Data | 78 (35)  72 (33)  48 (22)  22 (10)  0 | 127 (41)  90 (29)  20 (7)  70 (23)  2 | <0.0001^b^ |
| **Employment status**, *n* (%)    Unemployed    Employed    Student/retired    Missing Data | 120 (55)  94 (43)  5 (2)  1 | 128 (42)  155 (50)  24 (8)  1 | 0.001^b^ |
| **Individual income**, *n* (%)  Under $20,000/year  Over $20,000/year  Missing Data | 116 (55)  96 (45)  8 | 129 (44)  163 (56)  17 | 0.019^b^ |
| **Tobacco smoking**, *n* (%)    Current    Past    Never | 90 (41)  55 (25)  75 (34) | 95 (31)  63 (20)  151 (49) | 0.003^b^ |
| **Substance use,** *n* (%)    Current    Past    Never    Missing Data | 44 (20)  57 (26)  119 (54)  0 | 52 (17)  66 (21)  190 (62)  1 | 0.2168^b^ |
| **History of homelessness**, *n* (%)    Missing Data | 103 (47)  0 | 112 (37)  3 | 0.02^b^ |

^a^Mann Whitney U test, ^b^Chi-square test
