# Supplementary material for: Concordance between self-report and six commonly used clinical estimates or serological measures: Insights from a Canadian healthy aging study
Source: PLoS One. 2026 Apr 8;21(4):e0346489. doi: 10.1371/journal.pone.0346489 (PMC13061170; doi:10.1371/journal.pone.0346489)
Supplement: S2 Appendix — (DOCX) [file pone.0346489.s002.docx]

**S2 Appendix.** Missing data in self-reported data and clinical estimates

| **Condition/Virus** | **Self-report (n)** | **Clinical estimate (n)** |
| --- | --- | --- |
| CKD WLWH | 0 | 12 |
| CKD women without HIV | 3 | 5 |
| Liver Disease WLWH | 3 | 13 |
| Liver Disease women without HIV | 5 | 8 |
| Depression WLWH | 2 | 12 |
| Depression women without HIV | 4 | 16 |
| PTSD WLWH | 2 | 5 |
| PTSD women without HIV | 4 | 6 |
| HCV WLWH | 3 | 3 |
| HCV women without HIV | 1 | 0 |
| HBV WLWH | 6 | 3 |
| HBV women without HIV | 1 | 0 |

WLWH: women living with HIV; CKD: chronic kidney disease; PTSD: post-traumatic stress disorder; HCV: hepatitis C virus; HBV: hepatitis B virus
