# Supplementary material for: Concordance between self-report and six commonly used clinical estimates or serological measures: Insights from a Canadian healthy aging study
Source: PLoS One. 2026 Apr 8;21(4):e0346489. doi: 10.1371/journal.pone.0346489 (PMC13061170; doi:10.1371/journal.pone.0346489)
Supplement: Attachment — Submitted filename: Response to Reviewers.docx [file pone.0346489.s004.docx]

Manuscript reference number: **PONE-D-25-43093**

Title: **Concordance between self-report and six commonly used clinical estimates or serological measures in health research**

Dear Dr. Ennio Polilli and reviewers,

Thank you for your thoughtful review of our manuscript. We revised the manuscript to reflect the reviewer’s suggestions and provide a point-by-point response to the comments below. Revised parts of the manuscript are highlighted within quotations in blue.

**Journal Requirements:**

When submitting your revision, we need you to address these additional requirements.

1. Please ensure that your manuscript meets PLOS ONE's style requirements, including those for file naming. The PLOS ONE style templates can be found at

<https://journals.plos.org/plosone/s/file?id=wjVg/PLOSOne_formatting_sample_main_body.pdf> and 
<https://journals.plos.org/plosone/s/file?id=ba62/PLOSOne_formatting_sample_title_authors_affiliations.pdf>

Thank you for bringing this to our attention. We have updated the manuscript in accordance with PLOS ONE guidelines.

2. Thank you for stating the following financial disclosure:

“The British Columbia CARMA-CHIWOS Collaboration (BCC3) has received funding support from the Canadian Institutes of Health Research (CIHR) project grants (PJT-162348, PJT-175006), Community-based research grant (CBR-170103), and Women’s Health and Mentorship grant (F19-05017); CIHR HIV Clinical Trial Network support (CTN 335) and pilot grant funding (CTNPT046, CTNPT050); UBC Partner Recognition Fund; UBC Community University Engagement Support Fund, and UBC Public Scholar Initiative; Simon Fraser University’s Community Engagement Initiative; and Michael Smith Foundation of Health Research (trainee award). AK received a salary support award from the Canada Research Chair Program. MCMM received salary support from Michael Smith Foundation of Health Research Health Professional investigator award. TP received funding from the UBC Four Year Doctoral Fellowship, UBC Centre for Blood Research, and Mitacs Globalink Graduate Fellowship. TAGL received funding from the UBC Edwin S.H. Leong Centre for Healthy Aging Summer Student Research Award.”

Please state what role the funders took in the study.  If the funders had no role, please state: "The funders had no role in study design, data collection and analysis, decision to publish, or preparation of the manuscript." If this statement is not correct you must amend it as needed.

Please include this amended Role of Funder statement in your cover letter; we will change the online submission form on your behalf.

Thank you for this comment. We have included this statement in our updated funding section.

3. We note that you have indicated that there are restrictions to data sharing for this study. For studies involving human research participant data or other sensitive data, we encourage authors to share de-identified or anonymized data. However, when data cannot be publicly shared for ethical reasons, we allow authors to make their data sets available upon request. For information on unacceptable data access restrictions, please see <http://journals.plos.org/plosone/s/data-availability#loc-unacceptable-data-access-restrictions>.

Before we proceed with your manuscript, please address the following prompts:

a) If there are ethical or legal restrictions on sharing a de-identified data set, please explain them in detail (e.g., data contain potentially identifying or sensitive patient information, data are owned by a third-party organization, etc.) and who has imposed them (e.g., a Research Ethics Committee or Institutional Review Board, etc.). Please also provide contact information for a data access committee, ethics committee, or other institutional body to which data requests may be sent.

b) If there are no restrictions, please upload the minimal anonymized data set necessary to replicate your study findings to a stable, public repository and provide us with the relevant URLs, DOIs, or accession numbers. Please see <http://www.bmj.com/content/340/bmj.c181.long> for guidelines on how to de-identify and prepare clinical data for publication. For a list of recommended repositories, please see <https://journals.plos.org/plosone/s/recommended-repositories>. You also have the option of uploading the data as Supporting Information files, but we would recommend depositing data directly to a data repository if possible.

Please update your Data Availability statement in the submission form accordingly.

Thank you for raising this important point regarding long-term data access. To ensure compliance with PLOS’ Data Availability Policy, we now provide a non-author institutional contact for data requests. Specifically, requests can be directed to the BCC3 email address ([bcc3@cw.bc.ca](mailto:bcc3@cw.bc.ca)), which is managed at the institutional level rather than by an individual author. The dataset will be securely stored on the institutional server, and a standard operating procedure has been established to ensure continuity of access regardless of changes in personnel. Our data availability statement is as follows:

**Data availability statement:**

The minimal dataset underlying the findings of this study cannot be made publicly available due to ethical and privacy restrictions related to participant confidentiality. A substantial proportion of the data is derived from individuals living with HIV, a highly stigmatized condition, and the dataset includes data from Indigenous participants, for whom principles of community consultation and governance apply prior to public data sharing. The data may be made available upon request through a non-author institutional point of contact at the BCC3 study office ([bcc3@cw.bc.ca](mailto:bcc3@cw.bc.ca)). Access will be granted in accordance with institutional ethics, privacy policies, and applicable Indigenous data governance principles. Procedures are in place to ensure continuity of access independent of the study authors.

4. One of the noted authors is a group or consortium: British Columbia CARMA-CHIWOS Collaboration (BCC3; CIHR CTN 335)

In addition to naming the author group, please list the individual authors and affiliations within this group in the acknowledgments section of your manuscript. Please also indicate clearly a lead author for this group along with a contact email address.

Thank you for this comment. The names of all individuals were listed in the acknowledgement section.

The acknowledgement on lines (337-343) reads:

*“In addition to the authors listed here, the BCC3 study team includes co-principal investigators Valerie Nicholson and Drs. Jason Brophy, Neora Pick, Allison Carter, Carmen Logie, Kate Salters, Mona Loutfy, Jerilynn Prior, and Joel Singer, as well as trainees/research staff Beheroze Sattha, Izabella Gadawska, Charity Mudhikwa, Julliet Kien Zama, Loulou Cai, Dr. Monika Kowatsch, Davi Pang, Melanie Lee, Marcela AP Silva, Shayda A Swann, Franceska Dnestrianschii, Amber R. Campbell, Vyshnavi Manohara, Sofia Levy, and Camille Valbuena.”*

5. Please include captions for your Supporting Information files at the end of your manuscript, and update any in-text citations to match accordingly. Please see our Supporting Information guidelines for more information: <http://journals.plos.org/plosone/s/supporting-information>.

Thank you for bringing this to our attention, the captions for Supporting Information files have been added to the end of the manuscript.

Lines (467-469):

***“S1 Appendix.*** *Demographic characteristics of women living with HIV and without HIV in the BCC3 study*

***S2 Appendix.*** *Missing data in self-reported data and clinical estimates”*

**Reviewers' comments:**

Reviewer's Responses to Questions

**Comments to the Author**

1. Is the manuscript technically sound, and do the data support the conclusions?

The manuscript must describe a technically sound piece of scientific research with data that supports the conclusions. Experiments must have been conducted rigorously, with appropriate controls, replication, and sample sizes. The conclusions must be drawn appropriately based on the data presented.

Reviewer #1: Yes

Reviewer #2: Yes

Reviewer #3: Partly

2. Has the statistical analysis been performed appropriately and rigorously?

Reviewer #1: Yes

Reviewer #2: Yes

Reviewer #3: Yes

3. Have the authors made all data underlying the findings in their manuscript fully available?

The [PLOS Data policy](http://track.editorialmanager.com/CL0/http:%2F%2Fwww.plosone.org%2Fstatic%2Fpolicies.action%23sharing/1/010f019b4de01f25-81eb844b-837a-41b5-b73c-1dc48afab0de-000000/DvbFMybyZPW42SjFOgxcVS-buUZ90NnkQxffTkdhXQs=243) requires authors to make all data underlying the findings described in their manuscript fully available without restriction, with rare exception (please refer to the Data Availability Statement in the manuscript PDF file). The data should be provided as part of the manuscript or its supporting information, or deposited to a public repository. For example, in addition to summary statistics, the data points behind means, medians and variance measures should be available. If there are restrictions on publicly sharing data—e.g. participant privacy or use of data from a third party—those must be specified.

Reviewer #1: No

Reviewer #2: Yes

Reviewer #3: No

4. Is the manuscript presented in an intelligible fashion and written in standard English?

PLOS ONE does not copyedit accepted manuscripts, so the language in submitted articles must be clear, correct, and unambiguous. Any typographical or grammatical errors should be corrected at revision, so please note any specific errors here.

Reviewer #1: Yes

Reviewer #2: Yes

Reviewer #3: Yes

5. Review Comments to the Author

Please use the space provided to explain your answers to the questions above. You may also include additional comments for the author, including concerns about dual publication, research ethics, or publication ethics. (Please upload your review as an attachment if it exceeds 20,000 characters)

Reviewer #1: 1. Summary of the research

This manuscript investigates the concordance between self-reported and clinically assessed prevalence of selected chronic conditions and latent viral infections among women living with and without HIV in British Columbia, Canada. Using data from the BCC3 cohort, the study compares self-reported diagnoses of chronic kidney disease, liver disease, depression, post-traumatic stress disorder, hepatitis B, and hepatitis C with clinical estimates derived from screening tools and serological testing.

The authors report that agreement between self-report and clinical/serological measures varies by condition, with near-perfect concordance for HCV but only slight to fair agreement for other conditions. Cohen’s kappa statistics are used to quantify agreement. Demographic characteristics of the cohort are described.

The manuscript highlights challenges in relying solely on self-reported data for epidemiological research and clinical management, especially for conditions with low concordance. The authors recommend integrating self-report and objective measures to improve prevalence estimates and suggest that future research should address methodological limitations, explore subgroup differences, and provide more actionable recommendations for improving concordance.

Overall, the study addresses an important gap in understanding the reliability of self-reported health data in women living with and without HIV.

To strengthen the manuscript, the authors need to provide more comprehensive and transparent reporting of agreement metrics and statistical methods, expand the interpretation of findings by exploring causes of concordance and discordance, integrate demographic and contextual factors into the discussion, critically appraise study limitations, and offer concrete, actionable recommendations for future research and practice.

My overall recommendation is that the manuscript needs major revisions to meet PLOS ONE standards for transparency and methodological rigor.


2. Examples and evidence


2.1. Major technical issues

2.1.1. Title

a. The title is somewhat generic and not easy to read with the phrase "six commonly used clinical estimates or serological measures". The authors should consider specifying the population studied, what is being studied in short, and the country context

Thank you for this comment, we appreciate the need to make the title relevant and specific for readers. We have updated the title as follows:

“Concordance between self-report and six commonly used clinical estimates or serological measures: insights from a Canadian healthy aging study”

2.1.2. Abstract

a. Study Design Not Specified: The abstract does not indicate the study design. The authors will need to specify the study type in the methods.

Thank you for bringing this to our attention. We have revised the methods section to specify the study design. The abstract and Methods now clarify that women enrolled in the BCC3 Study, an interdisciplinary cohort study, were included in a cross-sectional analysis conducted between 2020 and 2024.

The abstract methods (lines 46-51) now reads:

*“Women (aged ≥ 16 years residing in British Columbia) enrolled in the BCC3 Study, a prospective cohort, between 2020 and 2024 were included in the cross-sectional analysis. Self-reported prevalence of six conditions/viruses (chronic kidney disease, liver disease, depression, post-traumatic stress disorder, and hepatitis B and C viruses (HBV, HCV)), were compared to clinical estimates based on screening tools, and serology. Agreement was assessed via Cohen’s kappa.”*

b. Inconsistent Sample Sizes: There is a discrepancy in reported sample sizes between the abstract and the results section. The authors should clarify the final analyzed sample sizes, explain any exclusions or missing data, and ensure consistency throughout the manuscript.

Thank you for this comment. We have revised the abstract to ensure consistency with the Results section by reporting the final analyzed sample sizes, rather than the total enrolled sample prior to application of exclusion criteria as was present before. Details regarding exclusions and missing data are now clearly described in the study flow diagram and Supplementary Materials.

Lines (53-54) now reads:

***“****In both women with (n=220) and without HIV (n=309), clinical estimate-based prevalence of depression and PTSD was higher than self-reported prevalence (all p<0.001).*”

c. Ambiguous Interpretation of Cohen’s Kappa: The abstract uses the phrase “fair to none” to describe agreement, which is non-standard and potentially confusing. It is recommended to report actual kappa values (with 95% confidence intervals if possible) for each condition, or at least the range. If qualitative descriptors are used (e.g., “fair,” “almost perfect”), the authors could cite a recognized interpretive scheme (such as Landis & Koch) and clarify these are heuristic. They should avoid using “none” as a category.

Thank you for this comment. We have revised the abstract to remove the non-standard “none” and to align all qualitative agreement terminology with the Landis and Koch interpretive scheme, which is now cited in the Methods section. The descriptor has been replaced with “poor”, consistent with this framework, and agreement terminology is clarified as and interpretive scheme.

Lines (50-51 and 59-60) now reads:

*“Agreement was assessed via Cohen’s kappa.”*

*“In both groups, agreement between self-report and clinical estimate of prevalence was fair to poor for all conditions/viruses (all κ<0.4), except for HCV, for which the agreement was near perfect (κ>0.8).”*

d. Statistical Reporting Lacks Precision: P-values and kappa values are presented without confidence intervals or details on the statistical approach. The authors should consider adding 95% confidence intervals for kappa values and clarify how agreement was assessed.

Thank you for this comment. We have ensured that 95% confidence intervals for all Cohen’s kappa values are presented in **Table 1**, and the statistical approach used to assess agreement is described in detail in the Methods section, including the use of Cohen’s kappa to account for chance agreement and the interpretation framework applied. In the Abstract, we summarize agreement patterns using ranges rather than reporting individual kappa values and confidence intervals to maintain conciseness and readability, consistent with journal conventions, while directing readers to the full results table for detailed estimates.

e. Unclear Definition of “Clinical Estimate”: The term “clinical estimate-based prevalence” is used without specifying that these are derived from screening tools or serology, not clinical diagnoses. The authors may need to briefly clarify what constitutes a “clinical estimate” in this context.

Thank you for this comment. To clarify the meaning of *clinical estimate–based prevalence*, we have revised the Methods section to state that these estimates are derived from screening tools and serologic measures, rather than formal clinical diagnoses.

The revised text now reads (Lines 48-51):

“Self-reported prevalence of six conditions/viruses (chronic kidney disease, liver disease, depression, post-traumatic stress disorder, and hepatitis B and C viruses [HBV, HCV]) was compared with clinical estimates based on screening tools and serology.”

f. Abstract Conclusions Too General: The conclusions are vague (lines 62-63 and lines 64-65). The authors could be more specific, avoiding use of “may”, and indicating which conditions showed the greatest discordance and the practical implications for researchers and clinicians.

Thank you for this comment. We acknowledge the reviewer’s suggestion to increase specificity in the Abstract conclusions. However, we intentionally retained a conservative level of interpretation in the Abstract to avoid overstatement of findings derived from a cross-sectional analysis. Given the condition-specific and context-dependent nature of concordance observed in this study, we aimed to summarize overall patterns rather than enumerate individual conditions or detailed implications in the Abstract. Another consideration was the limited word count allowed for this section, while greater specificity regarding which conditions demonstrated the greatest discordance, as well as the practical implications for researchers and clinicians, is provided in the Results, Discussion, and Conclusion sections, where sufficient detail and context are available to support interpretation.

g. Missing Keywords: The listed keywords do not include some important methodological terms. To improve the manuscript’s discoverability and better reflect its content, it is suggested that the authors consider adding such terms, including “concordance,” “agreement,” and “Cohen’s kappa”.

Thank you for this comment, we appreciate the opportunity to improve the manuscripts discoverability. We have determined the keywords using the MeSH on Demand tool (https://meshb.nlm.nih.gov/MeSHonDemand). We have updated the keywords using these tools and the reviewers suggestions:

*“HIV, female, self report, cohort studies, prevalence, comorbidity, cross-sectional studies, concordance”*

2.1.3. Introduction

a. Clarity and Specificity of Study Rationale: The introduction provides a broad background on the health disparities faced by women living with HIV compared to men, and the importance of accurate prevalence estimates while the study assesses concordance of self-reports vs clinical estimates or serology among women living with HIV vs those without HIV. It does not clearly articulate the specific gap in the literature regarding the concordance between self-report and clinical/serological measures for the selected conditions among women living with HIV or without HIV, nor does it explain why this comparison is particularly important for the study population. I would therefore recommend that the authors revise the introduction to address these issues.

Thank you for this comment. We have revised the Introduction to articulate the specific gap in the literature, noting that few studies have examined the concordance between self-reported concurrent conditions and clinical or serologic measures among women, either living with or without HIV. We also clarified why this comparison is particularly important for the study population by emphasizing that, among women living with HIV, discrepancies between self-reported and clinically derived data may reflect gaps in health literacy and patient–provider communication- improving awareness of diagnoses may support informed decision-making, treatment adherence, and ultimately quality of life.

Lines 91-100 of the introduction now reads:

*“In cohorts of people living with HIV, previous work has reported fair-to-moderate agreement for several comorbid conditions when comparing self-report with clinical or administrative data (17,18). Such discordance can compromise the accuracy of prevalence estimates, which can in turn influence the effectiveness of health-promoting interventions and research findings informed by self-reported data. In the context of women living with HIV, discrepancies in self-reported data can signal opportunities for health literacy promotion and improved provider-patient communication. Ensuring that women are aware of their diagnoses helps them make informed decisions and can positively affect treatment adherence and ultimately quality of life. However, few studies have systematically examined agreement across multiple physical and mental health conditions among women living with and without HIV using a consistent analytic framework.”*

b. Justification for Use of Cohen’s Kappa: The introduction does not mention the methodological approach (i.e., use of Cohen’s
kappa or other concordance metrics) or why it is appropriate for the research question. It is suggested that the authors briefly introduce the concept of concordance/agreement and the rationale for using kappa statistics to assess the alignment between self-report and clinical/serological measures. They should also consider referencing methodological challenges (e.g., the “kappa paradox,” prevalence effects) and why robust agreement metrics are needed.

Thank you for this comment. We recognize the importance of justifying our methodological approach and have done so as follows (Lines 105-108):

“*As simple percent agreement does not account for chance concordance, Cohen’s kappa (κ) was used to assess chance-corrected agreement for binary categorical variables. Although κ can be influenced by condition prevalence, it remains a standard and appropriate metric for evaluating concordance between self-reported and clinically derived health measures when interpreted in context.”*

c. Insufficient Context from Prior Literature: While the introduction mentions general discrepancies between self-report and clinical data and that studies comparing self-report to clinical estimates are “scarce”. It however does not sufficiently reference prior studies that have specifically examined concordance using kappa or similarm metrics, especially in HIV or similar populations. The authors should consider citing and briefly summarizing key findings from previous studies that have assessed agreement between self-report and clinical/serological data and highlight how this study builds on or differs from these prior studies.

Thank you for this comment. We have revised the Introduction to situate our study within the existing literature on concordance between self-reported and clinically derived health data. The revised text now cites prior studies using Cohen’s kappa and related agreement metrics, summarizing key findings that agreement tends to be higher for serologically confirmed infections and lower for mental health and chronic conditions, particularly in socially marginalized populations. We also reference HIV-specific studies that have reported fair-to-moderate agreement between self-reported and clinical or administrative data for several comorbid conditions. We also clarify how our study extends this literature by systematically examining agreement across multiple physical and mental health conditions among women living with and without HIV using a consistent analytic framework. These additions strengthen the contextual grounding of our work and clarify its contribution relative to prior studies.

Lines 89-93 now read:

*“Prior studies using Cohen’s kappa and related agreement metrics have demonstrated that agreement tends to be higher for serologically confirmed infections and lower for mental health and chronic conditions, particularly in marginalized populations (14–16). In cohorts of people living with HIV, previous work has reported fair-to-moderate agreement for several comorbid conditions when comparing self-report with clinical or administrative data (17,18).”*

d. Ambiguity in What Is Being Compared: The Introduction refers to “self-report-based prevalence” and “clinical estimates” but does not clarify how “clinical estimates” are derived vis a vis the screening tools or serology. The authors could clearly define in the introduction how “clinical estimates” are derived to the ensure readers can more easily interpret the results and limitations.

We appreciate this comment and agree that greater clarity was needed. We have revised the Introduction to define *clinical estimates* as prevalence measures derived from **screening tools and/or serologic data**, rather than clinician-adjudicated diagnoses. This clarification was added to help readers more easily interpret the comparisons made in the study and understand the associated limitations.

Lines 86-89 now read:

“*However, self-report data in the general population can be inconsistent with registered medical data and clinical estimates derived from screening tools and/or serology, producing over- or under-representation, depending on the condition and cohort studied (12,13).”*

e. Objective Statement Needs More Precision: The objective is not very specific. The authors could make it make it more precise by specifying the conditions/viruses being compared, the population, and the methodological approach.

Thank you for this comment. We have revised the objective statement to improve precision by specifying the conditions and viruses assessed, the study population, and the methodological approach.

The revised objective now reads (Lines 101-104):

*“Thus, the objective of this study is to assess agreement between self-reported and clinical or serological measures of chronic kidney disease, liver disease, depression, post-traumatic stress disorder, hepatitis C virus, and hepatitis B virus among women living with and without HIV enrolled in the British Columbia CARMA–CHIWOS (BCC3) Study, using Cohen’s kappa.”*

2.1.4. Methods

a. Study Design Not Indicated: As indicated in the abstract comments, the methods do not specify the study design, as required in the STROBE checklist. For transparency and context, authors should explicitly specify the study design in the title, abstract and methods of the manuscript.

Thank you for this comment, we have made efforts to state the study design in the abstract as mentioned above and the methods which now reads (Lines 112-113):

“This study used a cross-sectional analysis conducted within the British Columbia CARMA–CHIWOS (BCC3) cohort study.”

b. The authors’ use of the term “outcome” in the abstract and methods for describing chronic conditions and infections measured at baseline is not ideal as it typically refers to results or endpoints following an intervention or exposure. The authors should consider using alternative terms to improve clarity and align with standard epidemiological terminology, ensuring readers understand that the study is assessing prevalence rather than outcomes of an intervention.

Thank you for bringing this to our attention, we understand that outcomes may misrepresent our study design and have changed this terminology to “conditions” throughout.

c. Incomplete Reporting of Operational Definitions and Cutoffs: The methods section refers to validated tools (KFRE, FIB-4, CES-D-10, PLC-C) but does not specify the exact cutoffs or criteria used to define a positive case for each condition. The authors could explicitly state the thresholds or scoring criteria for each tool with references for reproducibility and transparency.

Thank you for this comment, we appreciate the need to discuss exact cutoffs to improve the reproducibility of our study and transparency of the methods. We have included a detailed breakdown of the assessment questions and criteria used in the study in **Fig 1.** We have also referenced the specific tools in the Methods section (lines 129-139):

*“Our self-report questions inquire about conditions diagnosed by a care provider rather than a perception of having a condition. These include CKD, liver disease, depression, PTSD, hepatitis C virus (HCV), and hepatitis B virus (HBV). Self-reported conditions are determined through survey responses, whereas clinical estimates are derived using a combination of survey data and validated assessment/screening tools: the Kidney Failure Risk Equation (22) and albuminuria levels (23) for CKD, the FIB-4 index (24–26), for liver disease, the 10-item Center for Epidemiologic Studies Depression Scale (CES-D-10) (27) for depression, and the 6-item PTSD Checklist-civilian version (PLC-C) (28) for PTSD, applying established cutoff values. Prevalence of chronic/latent viral infections is assessed using serological testing at the BC Centre for Disease Control. These included anti-HCV and anti-HBVc total (antibody against the hepatitis B core antigen).”*

***“Fig 1.*** *(a) Participant selection flowchart. (b) Self-report and clinical estimate assessment parameters for study condition/hepatitis viral infections. BCC3: British Columbia CARMA-CHIWOS Collaboration; PTSD: post-traumatic stress disorder; PLC-C: PTSD Checklist-civilian version; CES-D-10: Center for Epidemiologic Studies Depression Scale; KFRE: Kidney Failure Risk Equation; eGFR: estimated glomerular filtration rate; FIB-4: Fibrosis-4; HCV: hepatitis C virus; HBV: hepatitis B virus”*

d. Lack of Sample Size Justification or Power Consideration: There is no mention of how the sample size was determined, or whether the study is sufficiently powered to detect meaningful differences in kappa or prevalence. The authors may consider including a brief rationale for the sample size, even if post hoc, or discuss the precision of kappa estimates.

We thank the reviewer for this comment. As this study was conducted within an established cohort, the sample size was determined by participant availability rather than power calculations. To address concerns regarding precision, we report 95% confidence intervals for all Cohen’s κ estimates, which provide an indication of estimate stability and uncertainty.

We have added text to the Discussion acknowledging that precision varies by condition prevalence and that future studies may benefit from larger samples for low-prevalence conditions (lines 285-287):

*“The magnitude of this bias is expected to vary by condition, with greater impact for low-prevalence or asymptomatic conditions and those requiring longitudinal assessment.”*

e. Issues with Kappa Statistic Reporting and Interpretation: The Methods state that “agreement was assessed using Cohen’s kappa” but do not specify whether unweighted or weighted kappa was used, nor the rationale for the choice. There is also no mention of reporting 95% confidence intervals for kappa, which is necessary for robust interpretation, and the interpretation framework for kappa (e.g., Landis & Koch) is not specified. The authors could clarify whether kappa was unweighted or weighted and justify the choice, and they should consider including 95% CIs for rigorous statistical reporting for reproducibility and appraisal. They should also consider pre-specifying the interpretive framework for kappa values (e.g., Landis & Koch) and justify its appropriateness for their context. Furthermore, the authors could consider reporting observed and expected agreement (p₀, pₑ) and prevalence/bias indices.

Thank you for this comment. We agree that greater clarity regarding the assessment and interpretation of agreement was warranted. We have revised the Methods and Results sections to address these points.

We have added a reasoning for the use of unweighted kappa in our analysis, reporting of 95% confidence intervals, and reference to the interpretive scheme used (Landis & Koch). This section now reads (lines 156-160):

*“Agreement is assessed using unweighted Cohen’s kappa (κ), as agreement was evaluated between two binary categorical measures (presence vs absence) and a recognized heuristic interpretive scheme (22). Cohen’s kappa and corresponding 95% confidence intervals were calculated in GraphPad Prism (version 10.2.3) using equations 18.16 to 18.20 from Fleiss, Statistical Methods for Rates & Proportions (23).”*

f. Ambiguity in Statistical Testing for Paired Data: The authors mention use of chi-squared and Fisher’s exact tests for categorical variables, but do not clarify what test was used for paired binary data (self-report vs. clinical estimate for the same individual). The authors could therefore specify the statistical test used for comparing paired proportions.

Thank you for this comment. We did not perform hypothesis testing of paired proportions between self-reported and clinical estimate–based classifications at the individual level. Rather, our approach treated self-reported and clinical estimate–based prevalence as population level estimates, which were compared using chi-squared or Fisher’s exact tests, as appropriate, to assess differences in overall prevalence rather than within-individual change.

g. Software Version and Reproducibility: The methods mention GraphPad Prism 10.2.3, but do not specify the exact procedures or code used for kappa calculation and CIs. The authors could therefore briefly describe the procedure or provide a reference to the software’s method for kappa and CI calculation.

Thank you for this comment, we have added the following to better specify the exact code used for analyses (lines 158-160):

*“Cohen’s kappa and corresponding 95% confidence intervals were calculated in GraphPad Prism (version 10.2.3) using equations 18.16 to 18.20 from Fleiss, Statistical Methods for Rates & Proportions (23).”*

2.1.5. Results

a. Incomplete and Unclear Reporting of Agreement Metrics: The results section focuses on Cohen’s kappa and p-values but omits other important agreement metrics such as percent agreement, positive/negative agreement, and confidence intervals for kappa. These additional metrics are essential for interpreting the magnitude and reliability of agreement, especially when prevalence is skewed. To improve, the authors should consider also reporting percent agreement and, where appropriate, positive and negative agreement for each condition and group. They could also include 95% confidence intervals for all kappa statistics and briefly comment on the impact of prevalence and bias on kappa values to enhance interpretability.

We thank the reviewer for this helpful suggestion. In response, we have revised the Results to provide a more complete and interpretable assessment of agreement. Specifically, we now report overall percent agreement alongside Cohen’s kappa with 95% confidence intervals for each condition and group in the **Table 1**. In addition, we have expanded the Results text to describe agreement patterns narratively, including consideration of positive and negative agreement for the most methodologically informative conditions, particularly those with low prevalence or asymmetric marginal distributions. We also briefly comment on the impact of skewed prevalence and marginal asymmetry on kappa values to aid interpretation.

This is described in lines 187-195:

*“As skewed prevalence and marginal asymmetry can attenuate Cohen’s κ despite high observed agreement, percent agreement and condition-specific positive and negative agreement are used to contextualize agreement patterns where appropriate. For chronic kidney disease, agreement was largely driven by concordant negative classifications, reflecting low condition prevalence rather than strong concordance in positive identification between self-report and clinical estimate. For liver disease, high overall agreement primarily reflected negative agreement, with relatively fewer concordant positive cases contributing to attenuation of Cohen’s κ. For hepatitis B, skewed prevalence and higher clinical estimate based prevalence attenuated Cohen’s κ despite high negative agreement, indicating systematic differences between data sources impacting concordance.”*

b. Table 1: The table reports p-values and Cohen’s kappa values but does not provide confidence intervals for kappa, nor does it specify which statistical tests were used for each comparison. Qualitative descriptors for kappa are used without referencing a standard interpretive scheme. It is suggested that the authors revise the table to include 95% confidence intervals for all Cohen’s kappa values in the table, clearly indicate which statistical test was used for each p-value (e.g., Mann-Whitney U, chi-square, Fisher’s exact) and reference a recognized interpretive scheme for kappa descriptors (e.g., Landis & Koch) in the table legend or footnotes.

Thank you for this comment. We have revised **Table 1** to include 95% confidence intervals for all Cohen’s κ estimates, specified the statistical test used for each p-value, and referenced the interpretive scheme used for κ descriptors (Landis and Koch) in the table footnote. These revisions were made to improve clarity, transparency, and reproducibility of the reported agreement metrics.

c. Readability and Transparency in Statistical Reporting: The results are densely presented, with minimal narrative synthesis and unclear statistical methods or handling of missing data. To improve clarity, the authors could summarize key findings in a concise narrative, specify the statistical tests used and any p-value adjustments, and clearly state how missing data were handled and their potential impact on the findings.

Thank you for this comment. The Results section was intentionally structured to present the data in a concise and neutral manner, with minimal interpretation, in keeping with standard reporting conventions. All statistical tests, including the rationale for test selection and p-value handling, are described in the Methods section to avoid redundancy and maintain clarity between analytic procedures and results. In response to this comment, we have added brief narrative signposting to guide the reader through key findings and have included a statement describing the extent of missing data.

Line 180 reads: *“Missing data are described in* ***Supplement Table 2*** *(≤3% for all variables).”*

d. Lack of Sensitivity or Subgroup Analyses: No sensitivity or subgroup analyses are reported, which are recommended when prevalence is skewed, or agreement differs by subgroup. If feasible, the authors should consider reporting sensitivity analyses using alternative agreement indices and presenting agreement metrics stratified by key subgroups such as age or ethnicity. If the authors do not perform such analyses, they may need to acknowledge this as a limitation and suggest future research.

Thank you for this suggestion. While sensitivity and subgroup analyses were not conducted in the present study, we have acknowledged this as a limitation and revised the Discussion accordingly.

Specifically, we added the following statement to the Limitations section (Lines 293-296):

*“An additional limitation is the absence of sensitivity analyses using alternative agreement indices or subgroup-specific analyses (e.g., by age or ethnicity), which may be informative in the presence of skewed prevalence and should be explored in future studies.”*

2.1.6. Discussion

a. Limited Analysis of Concordance and Discordance Across Conditions: The discussion summarizes the main findings but does not sufficiently analyze why certain conditions (e.g., HCV) show strong agreement between self-report and clinical/serological measures, while others (e.g., depression, PTSD, HBV) do not. To improve, the authors could expand the discussion to explore possible mechanisms for condition-specific differences in concordance, considering psychosocial, healthcare system, and other relevant factors. Comparing the magnitude of kappa values and prevalence discrepancies, and highlighting similarities and differences, would provide a more nuanced interpretation and better contextualize the findings.

We thank the reviewer for this comment. In response, we have expanded the Discussion to provide a more detailed, condition-specific interpretation of concordance and discordance observed across the study conditions. Specifically, we now contrast conditions with near-perfect agreement (e.g., HCV) with those demonstrating lower agreement (e.g., depression, PTSD, and HBV), and discuss possible explanations for these differences. These include variation in screening and diagnostic practices, differences between symptom-based screening tools and participant awareness of formal diagnoses, the asymptomatic nature of certain infections, and the potential influence of stigma and patient–provider communication on self-report accuracy.

Lines 227-229 and 245-246 now read:

*“Lower agreement for depression and PTSD may reflect differences between symptom-based screening tools and participants’ understanding or recall of formal diagnoses, as well as stigma surrounding mental health conditions”*

*“Discordance may arise from limited awareness of prior exposure detected through serology, particularly when infection was asymptomatic or resolved without clinical diagnosis”*

b. Insufficient Integration of Demographic Characteristics, Generalizability, and Literature: Although demographic characteristics are reported in the results, they are not explicitly used in the discussion to assess generalizability or explore sources of bias or variation in concordance. Additionally, engagement with the broader literature on concordance between self-report and clinical/serological measures is limited. I would recommend that the authors discuss how demographic factors (e.g., age, ethnicity, socioeconomic status, substance use) may influence self-report accuracy and concordance, reflect on the applicability of the findings to other populations, and expand the literature review to include more studies on concordance, especially in similar populations.

Thank you for this comment. In response, we have revised the Discussion to integrate socio-demographic context and relevant literature into the interpretation of concordance patterns. We now discuss how socioeconomic disadvantage, substance use, and histories of homelessness (prevalent in both study groups) may influence health literacy, access to care, recall of diagnoses, and willingness to disclose health conditions, all of which can affect the accuracy of self-reported data. We also reference prior studies demonstrating that concordance between self-report and clinical or serological data varies by demographic and social factors, particularly among women and marginalized populations. We have also clarified the implications for generalizability, noting that our findings should be interpreted within the socio-structural context of the cohort while remaining informative for similar populations.

These additions are under the “Contextual Factors Affecting Concordance” subheading on lines 256-263:

*“Although subgroup analyses were not conducted, the socio-demographic context of this cohort is important for interpreting concordance patterns. High levels of socioeconomic disadvantage, substance use, and histories of homelessness in both groups may influence health literacy, access to care, and recall of diagnoses, all of which can affect the accuracy of self-reported data (14,16). Prior studies have similarly shown that concordance between self-report and clinical data varies by demographic and social factors, particularly among women and marginalized populations (3,15,16). While our findings may not generalize to populations with different socio-structural contexts, they underscore the importance of interpreting self-report data within the lived realities of the populations studied.”*

c. Insufficient Integration with Broader Literature: The discussion references a few studies to contextualize findings but overall, it lacks a deeper engagement with the broader literature on concordance between self-report and clinical/serological measures. I would suggest that the authors expand the literature review to include more studies on self-report vs. clinical/serological concordance, especially in similar populations (e.g., women, people living with HIV, marginalized groups).

We appreciate this comment and have expanded the Discussion to more fully engage with the broader literature on concordance between self-reported and clinically derived health data. Specifically, we now cite studies demonstrating that agreement varies across conditions and populations, with lower concordance often observed for stigmatized, asymptomatic, or symptom-based conditions and among women and socially marginalized groups. We also contextualize our findings on hepatitis B and C within existing literature on serologic detection, screening practices, and disclosure dynamics. These changes are within the “Contextual Factors Affecting Concordance” subheading.

d. Superficial Treatment of Study Limitations and Methodological Constraints: The limitations section acknowledges some constraints but does not sufficiently address potential biases (e.g., selection bias, recall bias, social desirability bias) or discuss their impact on the interpretation of findings. The authors could provide a more nuanced discussion of potential biases, including how participant selection and self-reporting may affect generalizability and internal validity. They could also discuss the possible direction and magnitude of bias introduced by these limitations and suggest methodological improvements for future studies, such as longitudinal designs, validation against medical records, and inclusion of additional conditions.

Thank you for this comment. We have revised the Limitations section to provide a more detailed discussion of potential sources of bias and their implications for interpretation. In particular, we now clarify how reliance on cross-sectional clinical estimates rather than confirmed medical diagnoses may affect internal validity, especially for conditions that require serial assessment or may change over time. We also discuss recall bias, social desirability bias, and stigma-related underreporting associated with self-reported data, and describe the likely direction of bias, noting that these factors are expected to attenuate observed agreement rather than inflate concordance, with the magnitude of bias varying by condition.

In addition, we acknowledge the potential impact of care engagement and cohort participation on generalizability, noting that a substantial proportion of women living with HIV in our study were connected to a specialized women-centred HIV care clinic, which may limit extrapolation to populations facing different socio-structural challenges. We further note the absence of sensitivity and subgroup analyses as a limitation and identify methodological priorities for future research, including validation against clinical diagnoses, longitudinal study designs, and inclusion of additional clinically relevant conditions.

Lines 285-301 now read:

*“These biases are likely to skew estimates toward underreporting, thereby attenuating observed agreement between self-reported and clinically derived measures rather than inflating concordance. The magnitude of this bias is expected to vary by condition, with greater impact for low-prevalence or asymptomatic conditions and those requiring longitudinal assessment. Importantly, 71% of women living with HIV in our study are connected to the Oak Tree Clinic, a specialized centre practicing comprehensive women-centred HIV care (40). While observations in this analysis appeared to be condition-specific, rather than associated with HIV status, suggesting that the care site does not necessarily affect the findings, we acknowledge that our findings may not be generalizable to populations facing different socio-structural challenges than our cohort participants. An additional limitation is the absence of sensitivity analyses using alternative agreement indices or subgroup-specific analyses (e.g., by age or ethnicity), which may be informative in the presence of skewed prevalence and should be explored in future studies. Lastly, we were not able to include some conditions that are highly relevant for women with HIV, such as lung disease, osteoporosis, dyslipidemia, or cardiovascular disease. Given these limitations, future studies should validate self-reported and clinically derived measures against confirmed clinical diagnoses and adopt longitudinal designs to better characterize concordance over time. Where feasible, linkage to medical records and evaluation of health literacy interventions may help identify and reduce sources of discordance.”*

e. Lack of Practical Implications and Specific Recommendations for Future Research and Practice: The discussion does not sufficiently address the practical implications of the observed concordance or discordance for epidemiological research, clinical management, or public health policy, particularly in marginalized populations. Recommendations for future research are somewhat generic and do not provide concrete suggestions for improving concordance or addressing identified gaps. The authors should elaborate on how their findings could inform future research design, clinical screening practices, and public health interventions, and offer specific recommendations for improving concordance and addressing the limitations identified.

We thank the reviewer for this comment. In response, we have revised the Discussion to articulate the practical implications of observed concordance and discordance for epidemiological research, clinical practice, and public health, and to provide specific, actionable recommendations. We now emphasize that reliance on a single data source may misestimate prevalence when clinical charts are unavailable, and that integrating self-reported and clinically derived measures may offer a more accurate approach in observational studies involving women living with HIV. We also outline clinical implications, noting that discordance may highlight opportunities to strengthen communication around diagnoses and screening results, particularly for mental health conditions and asymptomatic infections. From a public health perspective, we discuss the relevance of targeted screening and knowledge-mobilization strategies that address stigma and structural barriers to disclosure.

In addition, we provide concrete directions for future research, including validation of self-reported and clinically derived measures against confirmed clinical diagnoses, adoption of longitudinal study designs to better characterize concordance over time, linkage to medical records where feasible, and evaluation of health literacy interventions to reduce sources of discordance.

These changes can be found under the “Implications and Future Directions” subheading on lines 303-312:

*“Our findings of suboptimal agreement between self-reported data and clinical estimates for several concurrent conditions suggest that integrating both types of data may be a more effective way to accurately gauge the prevalence of physical and mental concurrent conditions in observational studies enrolling women living with HIV, when clinical charts are unavailable. Clinically, discordance may highlight opportunities to strengthen communication around diagnoses and screening results, particularly for mental health conditions and asymptomatic infections. From a public health perspective, these findings support targeted screening and knowledge-mobilization strategies that address stigma and structural barriers to disclosure. Enhancing the concordance between self-reported and clinical data not only increases the utility of self-report-based HIV research, it also empowers women, thereby contributing to more effective public health.”*

2.1.7. Conclusion:

a. Overstatement of Findings: The conclusion overgeneralizes by stating that “women living with and without HIV in British Columbia are well-informed about their HCV status,” without adequately considering the study limitations. To authors should consider tempering the conclusion and explicitly acknowledging that concordance was lower for other conditions, and discussing the implications for research and clinical practice.

Thank you for this comment. We agree that the original conclusion overstated the findings by implying broad awareness of health conditions. We have revised the Conclusion to temper this language by clarifying that high concordance was observed for HCV specifically, while agreement between self-reported and clinically derived measures was lower and more variable for other conditions. In addition, we now briefly highlight the implications of this discordance for research and clinical practice, emphasizing the need for cautious interpretation of self-reported data and the value of integrating multiple data sources.

Lines 314-319 of the Conclusion now read:

*“In this cohort of women living with and without HIV in British Columbia, self-reported lifetime HCV history showed high concordance with serologic measures, whereas agreement for other physical and mental health conditions with clinical estimates was lower, highlighting opportunities to improve screening and communication for liver disease, kidney disease, depression, PTSD, and HBV. Such discordance may bias prevalence estimates in research, obscure unmet clinical needs, and complicate public health planning when self-reported data are used in isolation.”*

b. Insufficient Emphasis on Implications of Discordance: While the conclusion notes challenges in aligning self-reported and clinical data, it does not clearly articulate why discordance matters or its impact on research, clinical care, and public health. The authors should clarify the consequences of poor concordance and suggest how integrating self-report with clinical and serological data could enhance accuracy and utility in future studies and practice.

Thank you for this comment. We have revised the Conclusion to more clearly articulate why discordance between self-reported and clinically derived data matters and its implications for research, clinical care, and public health. The revised text now explains that poor concordance may bias prevalence estimates, obscure unmet clinical needs, and complicate public health planning when self-reported data are used in isolation. We also emphasize that integrating self-reported, clinical, and serological data may improve accuracy and utility in both research and practice, while acknowledging the complexity of factors underlying discordance beyond communication alone.

Lines 317-321 of the Conclusion now read:

“*Such discordance may bias prevalence estimates in research, obscure unmet clinical needs, and complicate public health planning when self-reported data are used in isolation. The alignment between self-reported data and clinical estimates presents challenges, and while enhancing health literacy could empower women, the factors influencing these discrepancies are more complex than communication alone.*”

c. Lack of Specific Recommendations:
The conclusion calls for “community-based knowledge mobilization” and “improving health literacy,” but lacks concrete, actionable recommendations for researchers, clinicians, or policymakers. The authors should provide specific guidance, such as strategies for improving concordance, recommendations for future research design, or targeted interventions to address identified gaps.

Thank you for this comment. We have revised the Conclusion to include actionable recommendations addressing the implications of discordance and identified gaps. The revised text now specifies concrete strategies for improving concordance, including integrating self-reported, clinical, and serological data, adopting longitudinal study designs in future research, and incorporating community-based knowledge mobilization to share findings with participating communities and support health literacy. We also clarify how these approaches may improve prevalence estimation, inform clinical care, and enhance public health planning.

Lines 322-325 of the Conclusion now read:

“Strategies to improve concordance include integrating self-reported, clinical, and serological data, adopting longitudinal study designs to assess concordance over time, and incorporating community-based knowledge mobilization to share findings with participating communities they serve and improve health literacy for both personal wellbeing and research accuracy.”

2.2. General comments (relevant to all sections)

a. Data Availability Statement Not PLOS ONE Compliant: The Data Availability statement (“data available upon reasonable request”) does not meet PLOS ONE’s requirement for public sharing of the minimal dataset. It is recommended that the authors consider addressing this issue.

Thank you for raising this important point regarding long-term data access. To ensure compliance with PLOS’ Data Availability Policy, we now provide a non-author institutional contact for data requests. Specifically, requests can be directed to the BCC3 email address ([bcc3@cw.bc.ca](mailto:bcc3@cw.bc.ca)), which is managed at the institutional level rather than by an individual author. The dataset will be securely stored on the institutional server, and a standard operating procedure has been established to ensure continuity of access regardless of changes in personnel. Our data availability statement is as follows:

**Data availability statement:**

The minimal dataset underlying the findings of this study cannot be made publicly available due to ethical and privacy restrictions related to participant confidentiality. A substantial proportion of the data is derived from individuals living with HIV, a highly stigmatized condition, and the dataset includes data from Indigenous participants, for whom principles of community consultation and governance apply prior to public data sharing. The data may be made available upon request through a non-author institutional point of contact at the BCC3 study office ([bcc3@cw.bc.ca](mailto:bcc3@cw.bc.ca)). Access will be granted in accordance with institutional ethics, privacy policies, and applicable Indigenous data governance principles. Procedures are in place to ensure continuity of access independent of the study authors.

b. Manuscript Structure and Flow: The manuscript’s overall structure and flow would benefit from greater focus and logical progression across all sections. Specifically:
o In the introduction, transitions between the background, knowledge gap, and study objectives are unclear. The authors could revise the introduction for better logical flow and clear transitions for smoother progression.
o The Results section is dense and would be improved by using clearer subheadings or bullet points to highlight key findings by condition.
o The discussion section does not transition well with regards to ideas about interpretation of findings, limitations and implications, which could make it difficult for readers to follow the argument and understand the significance of the results. The authors could better organize the discussion into well-defined themes with clear transitions, ensuring that each idea logically follows from the previous one to enhance readability and clarity.

Addressing these points throughout the manuscript will help guide the reader, enhance clarity, and align the work with PLOS ONE guidelines for scientific writing.

Thank you for this comment. We revised the manuscript to improve overall structure, focus, and logical flow across sections. In the Introduction, we clarified transitions between the background, identified knowledge gap, and study objective by reorganizing paragraphs to progress from sex- and gender-specific health disparities among women living with HIV, to the reliance on self-reported data, to prior concordance literature using Cohen’s kappa, and finally to the specific gap addressed by this study. Explicit transition language was added to guide the reader through this progression such as on lines 95 and 101:

“In the context of women living with HIV…” and “Thus, the objective of this study is to…”

In the Results, we improved readability by using clearer subheadings to distinguish demographic characteristics, agreement analyses, and prevalence comparisons, allowing key findings to be more easily identified by condition. In the Discussion, we reorganized content into well-defined thematic sections (principal findings; concordance for mental health and chronic physical conditions; concordance for chronic viral infections; contextual factors; limitations; and implications), with clearer transitions between interpretation, limitations, and implications to enhance coherence and narrative flow.

c. Language, Clarity and Conciseness: Some sentences in the manuscript are long and complex, which can hinder understanding—for example, those found in lines 108–110 and 184–187. Additionally, there is frequent use of passive voice, as seen in lines 113–114 and line 148. To enhance clarity, the authors could break up lengthy sentences, use straightforward and direct language, and favor active voice. The authors could ensure that each sentence communicates a single, clear idea, and they remove redundant phrases to maintain conciseness.

Thank you for this comment. We have revised the manuscript to improve clarity and readability by breaking up long or complex sentences, particularly in the sections highlighted (e.g., lines 108–110 and 184–187), so that each sentence conveys a single, clear idea. We also reduced passive voice in favor of more direct, active constructions where appropriate and standardized verb tense throughout the manuscript for consistency.

For instance, lines 115-120 (previously 108-110) now read: “Recruitment for the BCC3 study began in December 2020 and includes women living with HIV and socio-demographically similar women without HIV ≥16 years old in British Columbia, Canada. Eligible participants must be able to communicate in English, provide written informed consent, are not pregnant or breastfeeding, and are able to attend a clinical visit, usually at the BC Women’s Hospital in Vancouver, or during recruitment drives in Prince George, Victoria, and Surrey.”

Lines 219-222 (previously 184-187) now read:

*“Our study found that concordance between self-report and clinical estimates among women living with and without HIV was condition and virus specific. Self-reported lifetime HCV history showed high agreement with serologic measures, whereas agreement for other physical and mental health conditions was lower, with similar patterns observed regardless of HIV status.”*

d. Terminology Consistency: There is lack of consistency in the use of some terms in the manuscript. For example, the interchangeable use of the terms “concurrent conditions” and “comorbidities” throughout the manuscript may cause confusion. For greater consistency and clarity, the authors should select one term and use it uniformly. Additionally, qualitative descriptors for statistical measures like Cohen’s kappa should follow a recognized interpretive scheme and be applied consistently, ensuring readers can easily understand and compare results.

Thank you for this comment. We agree that consistent terminology is important for clarity. In response, we have reviewed the manuscript to ensure consistent use of terminology and have adopted “concurrent conditions” as the primary term throughout. We initially reference the term “comorbidities” in the Introduction to align with established literature, and then define and transition to “concurrent conditions,” reflecting feedback from women with lived experience in our study who emphasized the importance of person-centred language. This approach is now clearly explained at first use, and the term “concurrent conditions” is used consistently thereafter.

Lines 71-73 read:

*“Women living with HIV experience a greater burden of age-related comorbidities (hereafter concurrent conditions)…”*

In addition, we have ensured that qualitative descriptors for Cohen’s kappa are applied consistently throughout the manuscript and reference a recognized interpretive scheme (Landis and Koch) in the Methods and table footnotes to support transparency and comparability across results.

This is now outlined explicitly in the Abstract on lines 49-51, and in the Methods on lines 156-158:

*“Agreement was assessed via Cohen’s kappa applying the Landis & Koch interpretive scheme.”*

*“as agreement is evaluated between two binary categorical measures (presence vs absence) and a recognized heuristic interpretive scheme”*

Reviewer #2: This article is well written and emphasizes the need for enhancing health literacy and improving communication between women and their care providers.
The limits of the study are accounted for. The conclusions are based on the results.

I have minor comments that may be due to my misunderstanding, but I would not define as "fair" agreements with a Kappa score below 0.4 (for example lines 161-162), as in my knowledge it would be classified as "poor" or "weak".

We thank you for raising this point. We agree that terminology used to categorize kappa values varies across the literature and depends on the classification framework applied. In the revised manuscript, we have clarified our language to avoid potential ambiguity by reporting kappa estimates with 95% confidence intervals alongside percent agreement and interpretive scheme. Where descriptors are used, they now align with the Landis and Koch framework, under which kappa values between 0.21 and 0.40 are classified as “fair.” This revision improves clarity and ensures consistency with established interpretive conventions.

The interpretive framework used in this study is now referenced explicitly in the Abstract on lines 49-51, and in the Methods on lines 156-158:

*“Agreement was assessed via Cohen’s kappa applying the Landis & Koch interpretive scheme.”*

*“as agreement is evaluated between two binary categorical measures (presence vs absence) and a recognized heuristic interpretive scheme”*

Reviewer #3: The study sounds alright in the way it is presented. However, the major downside is the unavailability of access to primary data used in the research. I would highly recommend that the researchers do the needful to have approval rights for the audience to access the primary data. If the primary data contains sensitive client details, kindly blind such details and give your readers confidence into what you have analysed.

Thank you for raising this important point regarding long-term data access. To ensure compliance with PLOS’ Data Availability Policy, we now provide a non-author institutional contact for data requests. Specifically, requests can be directed to the BCC3 email address ([bcc3@cw.bc.ca](mailto:bcc3@cw.bc.ca)), which is managed at the institutional level rather than by an individual author. The dataset will be securely stored on the institutional server, and a standard operating procedure has been established to ensure continuity of access regardless of changes in personnel. Our data availability statement is as follows:

**Data availability statement:**

The minimal dataset underlying the findings of this study cannot be made publicly available due to ethical and privacy restrictions related to participant confidentiality. A substantial proportion of the data is derived from individuals living with HIV, a highly stigmatized condition, and the dataset includes data from Indigenous participants, for whom principles of community consultation and governance apply prior to public data sharing. The data may be made available upon request through a non-author institutional point of contact at the BCC3 study office ([bcc3@cw.bc.ca](mailto:bcc3@cw.bc.ca)). Access will be granted in accordance with institutional ethics, privacy policies, and applicable Indigenous data governance principles. Procedures are in place to ensure continuity of access independent of the study authors.
